# On-Chip Optical Nonreciprocity Using an Active Microcavity

**DOI:** 10.1038/srep38972

**Published:** 2016-12-13

**Authors:** Xiaoshun Jiang, Chao Yang, Hongya Wu, Shiyue Hua, Long Chang, Yang Ding, Qian Hua, Min Xiao

**Affiliations:** 1 National Laboratory of Solid State Microstructures, College of Engineering and Applied Sciences, and School of Physics, Nanjing University, Nanjing 210093, China; 2 Department of Physics, University of Arkansas, Fayetteville, Arkansas 72701, USA

## Abstract

Optically nonreciprocal devices provide critical functionalities such as light isolation and circulation in integrated photonic circuits for optical communications and information processing, but have been difficult to achieve. By exploring gain-saturation nonlinearity, we demonstrate on-chip optical nonreciprocity with excellent isolation performance within telecommunication wavelengths using only one toroid microcavity. Compatible with current complementary metal-oxide-semiconductor process, our compact and simple scheme works for a very wide range of input power levels from ~10 microwatts down to ~10 nanowatts, and exhibits remarkable properties of one-way light transport with sufficiently low insertion loss. These superior features make our device become a promising critical building block indispensable for future integrated nanophotonic networks.

Reciprocity, as framed by the Lorentz theorem^1^, is fundamental to light wave transport in linear, time-invariant optical systems with the preservation of time-reversal symmetry. Yet, optical signal processing and communications based on photonic integrated devices demand on-chip nonreciprocal light transmission, which is appealing even in theory because of the time-reversal symmetry held in light-matter interactions^2^,^3^. One way to break optical reciprocity is to operate the system in a nonlinear regime^4^,^5^,^6^ and harvest bistability. A more versatile approach is to direct light through a material which exhibits strong magneto-optical (Faraday) effect^7^,^8^. In practice, this effect has generally been adopted in commercial optical isolators and circulators (which are key components in today’s fiber-optics systems). However, this approach poses a critical challenge to the miniaturization of such components utilizing the current complementary metal-oxide-semiconductor (CMOS) technology, and the externally applied magnetic fields could influence the functionalities of nearby devices. By constructing a hybrid chip^9^,^10^, obtaining nonreciprocity with magneto-optical materials suffers from much increased fabrication complexities. Based upon the concept of interband photonic transitions^11^, an electrically driven symmetric device^12^ was reported in a silicon photonic integrated circuit. Although the concept is elegant and no hybrid technology is required, its implementation is so far complicated and the fabricated device displays over 70 dB insertion loss. In the emerging field of parity-time (PT) symmetric optics^13^, PT-symmetric media assembled with dissipation and gain components may allow one-way reflection^14^ under certain conditions. But in order to have a nonreciprocal transmission, Kerr-type nonlinearities^15^ are usually needed for breaching the time-reversal symmetry.

There are other reported observations on optical isolation by exploring electro-absorption modulation^16^, cholesteric liquid crystals^17^, and opto-acoustic effect^18^. However, these methods are incompatible with the conventional CMOS process. Despite most of the mechanisms for optical isolation could be used for the realization of optical circulators in principle, to date functional circulators have been mainly demonstrated with the traditional Faraday rotation using photonic crystal^19^,^20^ or microring resonators^21^ and Mach-Zehnder interferometers^22^. To be reconcilable with the existing CMOS technology, recent progresses were made on reducing the size of these circulators through resonant enhancement of the interaction between light waves and magneto-optically active media^21^,^22^. This generates the concepts for on-chip circulators using photonic-crystal^23^ or microring^24^,^25^,^26^ resonators. To date, there have been few experimental realizations for magneto-optical microring resonators^9^,^27^ but none for photonic-crystal microresonators. It is therefore intriguing to ask whether a simpler scheme, involving only existing components that are readily fabricated in the CMOS process, can be exploited to obtain optical nonreciprocity, isolation and circulation simultaneously in an on-chip silicon photonic platform.

In contrast with previous proposals, here we present a much simpler yet novel architecture employing only one active whispering-gallery-mode (WGM) microtoroid resonator to attain both on-chip optical isolation and (pseudo-) circulation functionalities with high-contrast asymmetric transmission (Fig. 1a–c). We notice that a passive resonant structure has been applied to enhance the thermo-optic nonlinearity in silicon, but its performance critically depends on the input light power^5^ (above 10 μW) and large power consumption. Albeit the compound system composed of two active-passive-coupled WGM microcavities^28^,^29^ allows asymmetric transmission, its complex structure brings up an uneasy task for precise controls of coupling strengths between composite subsystems. Moreover, its complexity increases insertion loss and makes large isolation to be difficult. As a comparison, our current scheme not only reduces simultaneously the insertion loss of the device, technological complexity and achievable footprint, but also allows a large range of tunable and switchable light nonreciprocity even when operating at ultralow input signal power levels. Thanks to the active cavity being utilized here, the output signal power can be larger than its original input because of the amplification. Limited by the power detection sensitivity of adopted photodetectors and insertion losses particularly in forward transmission measurements (Fig. 1d), we have realized ultra-low power optical isolation with an appreciable isolation ratio larger than 15 dB whereas the insertion loss of only few dB. Such a great isolation performance with sufficiently low insertion loss would be very appealing in other reported schemes^5^,^9^,^12^,^18^. As mentioned above, so far most demonstrations on on-chip three-port circulation rely on the Faraday effect. Interestingly, the illustrated three-port bidirectional transmission here could be considered as the first practical attempt to on-chip optical pseudo-circulation beyond the commonly adopted Faraday effect, and its successful demonstration could pave new avenues towards the development and applications of integrated silicon photonics.

## Results

**Figure 1:**
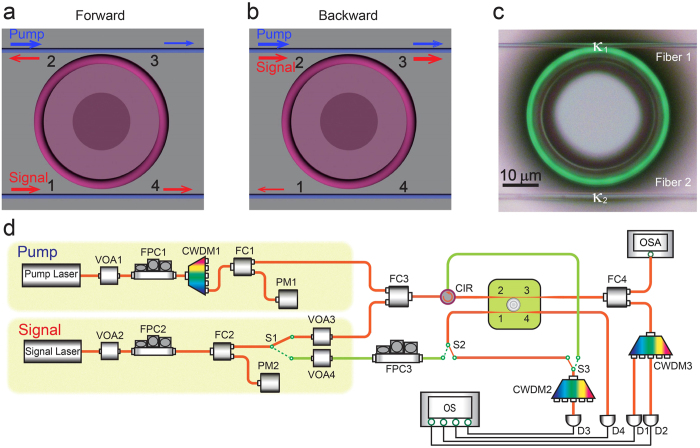
On-chip active whispering-gallery-mode (WGM) silica microtoroid resonator for optical isolation and circulation. (**a**) and (**b**) Schematic illustrations of forward and backward propagation configurations based upon signal input ports 1 and 2. (**c**) Top-view optical microscope image of the system in (**a**) or (**b**), which is composed of an active microtoroid cavity coupled to two tapered fibers (1 and 2) with coupling strengths *κ*_1_ and *κ*_2_. The erbium-doped microtoroid is pumped at the wavelength of 1480 nm band. **(d)** Schematic of the experimental setup. VOA, variable optical attenuator; FPC, fiber polarization controller; CWDM, coarse wavelength division multiplex; FC, optical fiber coupler; PM, power meter; S, optical switch; CIR, optical circulator; OSA, optical spectrum analyzer; OS, oscilloscope; D, photodetector.

Our nonreciprocal device is based on the strong gain-saturation nonlinearity existed in the active microtoroid cavity with high-quality (Q) factor, fabricated from an erbium-doped silica sol-gel film^30^,^31^,^32^. As schematically shown in Fig. 1a–c, the system is composed of a microtoroid coupled with two tapered optical fibers (labelled as fibers 1 and 2, respectively). The active microtoroid was optically pumped by a 1480 nm narrow-linewidth tunable laser to produce an effective gain (*g*) in the 1550 nm band. Here, the effective gain has the form of 
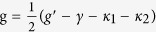
 with 
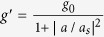
, where *g*′ is the real gain supplied by the doped Er^3+^ ions; *g*_0_ is the gain as the circulating signal-field amplitude inside the cavity becomes zero (i.e. *a* = 0); 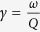 is the intrinsic decay rate caused by the cavity internal loss; *κ*_1_ (*κ*_2_) denotes the coupling strength between fiber 1 (2) and the microcavity; and *a*_*s*_ is the saturation amplitude at which *g*′ is half of *g*_0_. The cavity resonance is maintained at the 1550 nm band to coincide with the emission wavelength of erbium ions. To accurately tune the coupling strengths (*κ*_1_, *κ*_2_), both fiber tapers are mounted on nanopositioning translation stages for precision controls of their separation distances. The intrinsic Q-factors for the signal and pump wavelengths were measured to be 7.7 × 10^5^ at 1550.4 nm and 1.7 × 10^6^ at 1469.3 nm, respectively.

To catch the essential physics behind the functionalities of our device and without complicating the problem, we here present an intuitive picture by ignoring the scattering-induced mode splitting^33^. In the steady-state approximation, for the forward and backward propagation configurations (Fig. 1a,b), the output signal at ports 2 (1) and 4 (3) are, respectively, represented in terms of the signal field amplitude *a*_*F*,*B*_ inside the cavity for the forward (backward) configuration^34^,


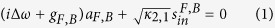


where Δ*ω *= *ω* − *ω*_0_ is the cavity detuning and 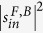 represents the incident signal power. The forward (backward) effective gain takes the form of 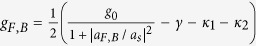. The normalized forward (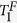) and backward (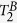) transmissions at ports 1 and 2 are given by


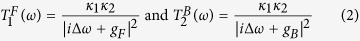


It is now apparent from (Eq. 2) that in order to break the time-reversal symmetry in the system and have asymmetric signal transmissions 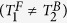, it requires asymmetrical couplings between the microcavity and the two fiber tapers (i.e. *κ*_1_ ≠ *κ*_2_). With the presence of the gain saturation nonlinearity, such asymmetric couplings will result in different *a*_*F*_ and *a*_*B*_, despite h*a*ving the same input signal power |S_in_|^2^. As a consequence, this results in the forward gain (*g*_*F*_) be distinct from backward gain (*g*_*B*_). Theoretically, by manipulating asymmetric geometrical couplings the system permits nonreciprocal transmission with high isolation ratio in a controllable manner. It is worth to emphasize that in the treatment above, both fiber tapers are assumed to be single mode. However, if high order modes were present, both measurements and theoretical analysis should be taken with caution^3^.

**Figure 2:**
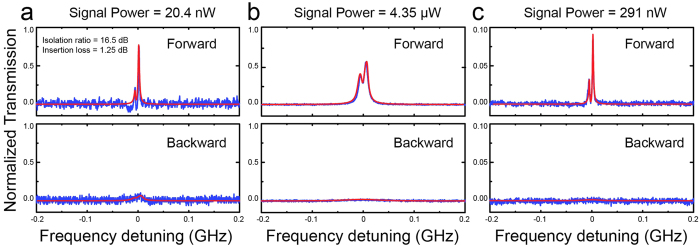
Superior optical-isolation performance of the system with remarkably low insertion loss. (**a**) Supersensitive nonreciprocal transmittance spectra at a sufficiently low signal power of 20.4 nW with an insertion loss of 1.25 dB. (**b**) Typical asymmetric transmittance spectra at the input signal power of 4.35 μW with an insertion loss of 2.70 dB. (**c**) Near perfect isolation where the backward transmission has no appreciable output signal. In (**a**) and (**b**) the isolation ratios are 16.5 dB and 17.1 dB, respectively. For figure (**c**), the parameters are: the dropped pump power of 31.0 μW, *κ*_1_ = 2*π* × 0.24 GHz and *κ*_2_ = 2*π* × 1.15 MHz. Hereafter, solid red curves are theoretical calculations.

**Figure 3:**
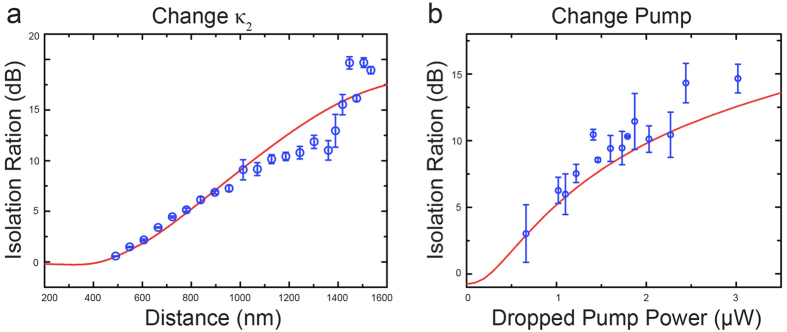
Optical isolation performance of the device. The isolation ratio as a function of the separation distance between the toroid and fiber 2 (**a**) or dropped pump power (**b**) with the same input signal power of 291 nW. Experimental data clearly indicate the capability of engineering the device performance by controlling different degrees of freedom of the system. Other parameters: (**a**) *κ*_1_ = 2*π* × 0.23 GHz; (**b**) *κ*_1_ = 2*π *× 0.24 GHz and *κ*_2_ = 2*π* × 1.15 MHz.

In order to test the device’s performance and controllability experimentally, we began with the investigation on the optical isolation (Fig. 1) by studying the backward and forward transmissions at ports 1 and 2. To start, the pump laser was first switched off and the system was reduced to a linear passive one. As expected, the outputs in the two directions yield nearly symmetric transmissions regardless of the difference between *κ*_1_ and *κ*_2_. Then, with the pump laser on, thanks to the high-Q factor, the circulating signal power inside the microresonator easily experiences gain saturation as the pump power is gradually increased. In the experiment, we have achieved the isolation ratios (defined as 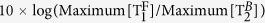 up to 20 dB for the input signal powers ranging from ~10 nW to ~10 μW with sufficiently low insertion losses (Figs 2 and 3), which outperforms most of the previously reported results as ascribed above. By optimizing the system parameters, the isolation ratio for the ultralow signal power can be further improved but subject to the power detection accuracy of available photodetectors (The noise level limits the measurable residue power in the *off* state). Moreover, the large tunable parameter ranges for *κ*_1_, *κ*_2_ and the dropped pump power provide additional useful knobs to engineer needed light nonreciprocity in such a device. For example, the supersensitivity of our device is illustrated in Fig. 2a where with the incident signal power of 20.4 nW, we have observed an isolation ratio of 16.5 dB with a very low insertion loss, 1.25 dB. As the signal power was increased to 4.35 μW, the system produces an isolation ratio of 17.10 dB with still a low insertion loss, 2.70 dB (Fig. 2b). Theoretically, near perfect one-way light transmission is possible under the extreme asymmetric coupling condition. Fortuitously, such perfect isolation has been evidenced in the experiment (Fig. 2c), where the backward transmission is almost halted (due to the very tiny transmitted signal power, the backward transmission is buried inside the detector noise background). The doublet structure appearing in Fig. 2a–c is caused by the scattering-induced mode splitting^33^. For fixed signal and pump laser powers, as well as a given *κ*_*1*_, the typical isolation behavior is plotted in Fig. 3a by steadily changing *κ*_*2*_ (i.e., altering the distance between the microcavity and fiber 2). The experimental data clearly verify our prediction that with a stronger asymmetric coupling geometry, the system yields larger asymmetric transmissions between forward and backward directions. Similarly, by fixing the input signal power and certain asymmetric coupling strengths, along with raising the dropped pump power, the device allows more forward transmission than backward and, therefore, leads to an increased isolation ratio (Fig. 3b). The changes of the insertion losses of our design in both cases are supplied in Supplementary Figures S2 and S3, where the isolation performance of the device is also examined as a function of the input power of the signal field.

**Figure 4:**
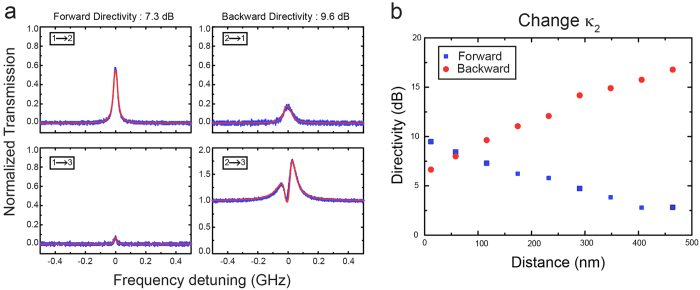
Optical circulation (i.e. bidirectional-transmission) performance. The device is formed by ports 1, 2 and 3 with the same input signal power of 312 nW, given *κ*_1_ = 2*π* × 0.85 GHz and the dropped pump power of 6.6 μW. (**a**) Typical nonreciprocal transmittance spectra with a forward directivity of 7.3 dB and a backward directivity of 9.6 dB. (**b**) Circulation performance characterized by the forward and backward directivities versus separation distance between the toroid and fiber 2.

For further evaluation of our nonreciprocal device, in the second part of this article, we describe how to realize a three-port optical pseudo-circulator in this system. With use of the same setting, we implemented three-port bidirectional transmission (as depicted in Fig. 1a,b) formed with ports 1, 2 and 3 (Port 4 is not of interest in the current work). For simplicity, we concentrate on the case where if the signal enters port 1 it emits from port 2; but if the signal is launched from port 2, it is emitted from port 3 (instead of being emitted from port 1). Experimentally, complying such a pseudo-circulator requires first the realization of high-contrast optical asymmetric transmission. By manipulating the coupling strengths *κ*_1_ and *κ*_2_, the system is then transformed to the optical circulation mode under certain parameter values with two figures of merit, sufficiently low insertion loss and high directivity (see Supplementary Figure S4). In our proof-of-principle demonstrations, the results shown in Fig. 4 clearly demonstrate the good performance of such a bidirectional-transmission device. Figure 4a shows its typical transmission spectra with high directivity in both directions. It is apparent that as the signal light is launched through port 1, the majority power is released from port 2 with a forward directivity of 7.30 dB, despite a small amount coming out of port 3 due to the scattering-induced back-reflection^33^; when the signal is launched from port 2, it is mainly emitted at port 3 with a backward directivity of 9.63 dB. Here, the Fano-like transmission from port 2 to port 3 in Fig. 4a is not due to the scattering-induced mode splitting, but the interference between the input signal field from fiber 1 and the instantaneous signal field dropped from the active microcavity (also see the Supplementary information). Although the isolation ratio is gradually increased by enlarging the separation distance between the fiber 2 and the microcavity (see Fig. 3a), the same conclusion cannot be directly applied to the optical bidirectional transmission. In contrast, as shown in Fig. 4b, the backward directivity monotonically follows the increased separation distance, but the forward directivity decreases. This observation suggests that the desirable optical circulation occurs not at the optimal isolation condition but at certain combined optimal forward and backward directivities. For the relationships between the directivities and dropped pump power, interestingly, we found that the optical pseudo-circulator (see Supplementary Figure S5) bears a similar behavior as for the asymmetric transmission (Fig. 3b). Both forward and backward directivities increase in a certain region and then start to saturate with further increase of the dropped pump power. Theoretical calculations (solid red curves in Figs 2, 3 and 4) that considering the dynamic gain in the active microcavity (for details, please refer to refs (28, 34 and 35, and elsewhere) and using the experimental parameters not only consistently match the measured transmission spectra, but also agree excellently with them, showing the well understanding of our system performance.

Very recently, we became aware of an interesting theoretical analysis^36^ in which the authors revealed a dynamic reciprocity behind nonlinear optical isolators based on Kerr or Kerr-type nonlinearities. The existence of this dynamic reciprocity sets the limits for applying Kerr or Kerr-type nonlinear optical isolators to laser protection. Despite the gain saturation would undergo the dynamic reciprocity if waves are launched from both forward and backward directions, our isolation scheme could still be useful for certain applications where fields are not desirable to be simultaneously present in both directions. In addition, the demonstrated pseudo-circulator above differs from a traditional circulator as it cannot provide directional light transport from port 3 to port 1 but not port 2 (Further discussion please refer to Supplementary information). We notice that the pseudo-circulator^37^ can be used to replace a beam splitter in an otherwise all-fiber system, because it is substantially more efficient and does not rely on free-space optics. To bypass the dynamic reciprocity, in a very recent work we have successfully realized a chip-based optical isolator based on direction-sensitive momentum conservation (or phase matching) in four-wave mixing parametric amplification occurring in a high-Q silica microtoroid^38^ for the first time. We note that by utilizing direction-sensitive phase matching, nonreciprocal optically induced transparency has been recently demonstrated in a high-Q microsphere cavity^39^.

## Discussion

In conclusion, we have accomplished nonreciprocal light transmission in the silicon photonic platform, which is expected to significantly impact on both fundamental research and device applications. As fundamental building blocks to obtain on-chip optical nonreciprocity, the demonstrated isolation and pseudo-circulation work in a broad operating scope for the input signal power ranging from ~10 nW to ~10 μW. The introduction of the gain-saturation induced nonlinearity here greatly suppresses the loss of the signal power. Similar to all optical devices through resonance enhancement, our system is subject to a limited bandwidth (about tens of MHz). To overcome the narrow bandwidth, one way is to exploit thermo-optic effect^5^ to tune the operating modes across a large wavelength range. It is worthwhile to point out that in the current classical regime, as the gain is supplied only for amplifying the cavity mode, the noise due to amplification plays a negligible role in isolation. More importantly, as a practical solution to integrate optical isolation and bidirectional transmission within the current CMOS process, our method of employing only one active microcavity extends a critical step and bypasses the common standard based upon the Faraday effect with sufficiently low insertion loss and excellent isolation performance for certain practical applications.

## Methods

### Sample Fabrication

For the fabrication of erbium-doped microtoroid cavities, first of all we prepared the erbium-doped silica film on a silicon chip using the sol-gel process as described in refs 30 and 31. To save fabrication time for making the erbium-doped silica film, we prepared the sol-gel film on a 500 nm thick pure thermal oxide film. By adding an 800 nm thick erbium-doped sol-gel film, the final thickness of the film is 1.3 μm. The average doping concentration of the erbium ions for the prepared film is about 2 × 10^19^ cm^−3^, after the preparation stage, the microtoroid cavities were then fabricated through combined processes of photolithography, buffered HF wet etching, XeF_2_ dry etching and CO_2_ laser reflow^30^.

### Optical isolation measurement

The experimental setup is schematically depicted in Fig. 1d for the optical nonreciprocal measurements, isolation and circulation. Two narrow linewidth tunable lasers, operating at the 1480 nm and 1550 nm bands, were chose as, respectively, the pump and signal light sources. Variable optical attenuators (VOA1 and VOA2) as well as fiber polarization controllers (FPC1 and FPC2) were used to regulate the input laser powers and polarizations. Two fiber couplers (FC1 and FC2) were chosen for the measurements of the pump and signal laser powers. The pump field, before entering FC1, traversed a coarse wavelength division multiplexer (CWDM1), filtering out the noise at the 1550 nm wavelength. Three optical switches (S1, S2 and S3) were employed to alter the paths of the signal light for measuring the power transmission difference between the forward and backward propagation configurations (see Fig. 1a,b; or Supplementary Figure S1). The use of optical switches allows us to measure the transmission difference between forward and backward transmissions without having to physically realign the setups between the input and output beam paths, as emphasized in the work by Jalas and his colleagues^3^.

For forward light transport measurements, the signal field was launched into the cavity through port 1 by connecting S1 and S2 to VOA4 and FPC3, as shown in Fig. 1d. Since the optical components and the optical fibers used in the experiment were not polarization maintained, here the use of FPC3 was to further adjust and ensure the same desired polarization for the signal light as it was launched from ports 1 and 2, respectively. The dropped signal light from port 2 was directed into an optical fiber circulator (CIR), a switch S3, and a CWDM2 (to separate out the reflected pump laser). Similarly, for backward light transport measurements, the signal beam was launched into the cavity through port 2 by connecting S1 to VOA3. The dropped signal light from port 1 was then directed into S2, S3 and CWDM2. The purpose of using the two variable VOA3 and VOA4 was for balancing the insertion losses in forward and backward transmissions as well as ensuring equal launching signal powers from both paths. The forward transmission in the experiment had more insertion loss than in the backward one, so we added certain additional loss using VOA4 to attain the same insertion losses for both transmission configurations. Besides, VOA3 is needed since each variable optical attenuator has its own insertion loss. We want to emphasize here that these arrangements are very important to ensure correct measurements for optical isolation as the gain saturation is very sensitive to the circulating signal power within the microcavity. CWDM3 was employed to separate the pump and signal power recorded by photodetectors D1 and D2, respectively^28^. It is worth pointing out again that it is important to maintain the same polarization of the signal light as its original input. In the experiment, we have observed that few dB isolation could be obtained simply due to the polarization effect.

## Additional Information

**How to cite this article:** Jiang, X. *et al*. On-Chip Optical Nonreciprocity Using an Active Microcavity. *Sci. Rep.* **6**, 38972; doi: 10.1038/srep38972 (2016).

**Publisher’s note:** Springer Nature remains neutral with regard to jurisdictional claims in published maps and institutional affiliations.

## Supplementary Material

Supplementary Information

## References

[b1] Potton R. J. Reciprocity in optics. Rep. Prog. Phys. 67, 717–754 (2004).

[b2] Barron L. D. Parity and optical activity. Nature 238, 17–19 (1972). 12635259 10.1038/238017a0

[b3] Jalas D. . What is - and what is not - an optical isolator. Nature Photon. 7, 579–582 (2013).

[b4] Soljačić M. & Joannopoulos J. D. Enhancement of nonlinear effects using photonic crystals. Nature Mater. 3, 211–209 (2004). 15034564 10.1038/nmat1097

[b5] Fan L. . An all-silicon passive optical diode. Science 335, 447–450 (2012). 22194410 10.1126/science.1214383 PMC5563475

[b6] Gallo K., Assanto G., Parameswaran K. R. & Fejer M. M. All optical diode in a periodically poled lithium niobate waveguide. Appl. Phys. Lett. 79, 314–316 (2001).

[b7] Dotsch H. . Applications of magneto-optical waveguides in integrated optics: review. J. Opt. Soc. Am. B 22, 240–253 (2005).

[b8] Levy M. Nanomagnetic route to bias-magnet-free, on-chip Faraday rotators. J. Opt. Soc. Am. B 22, 254–260 (2005).

[b9] Bi L. . On-chip optical isolation in monolithically integrated non-reciprocal optical resonators. Nature Photon. 5, 758–762 (2011).

[b10] Espinola R. L., Izuhara T., Tsai M.-C., Osgood R. M. & Dötsch H. Magneto-optical nonreciprocal phase shift in garnet/silicon-on-insulator waveguides. Opt. Lett. 29, 941–943 (2004). 15143634 10.1364/ol.29.000941

[b11] Yu Z. & Fan S. Complete optical isolation created by indirect interband photonic transitions. Nature Photon. 3, 91–94 (2009).

[b12] Lira H., Yu Z., Fan S. & Lipson M. Electrically driven nonreciprocity induced by interband transition on a silicon chip. Phys. Rev. Lett. 109, 033901 (2012). 22861851 10.1103/PhysRevLett.109.033901

[b13] Regensburger A. . Parity-time synthetic photonic lattices. Nature 488, 167–172 (2012). 22874962 10.1038/nature11298

[b14] Feng L. . Experimental demonstration of a unidirectional reflectionless parity-time metamaterial at optical frequencies. Nature Mater. 12, 108–113 (2012). 23178268 10.1038/nmat3495

[b15] Sukhorukov A. A., Xu Z. Y. & Kivshar Y. S. Nonlinear suppression of time reversals in PT-symmetric optical couplers. Phys. Rev. A 82, 043818 (2010).

[b16] Ibrahim S. K., Bhandare S., Sandel D., Zhang H. & Noe R. Non-magnetic, 30 dB integrated optical isolator in III/V material. Electron. Lett. 40, 1293–1294 (2004).

[b17] Hwang J. . Electro-tunable optical diode based on photonic bandgap liquid-crystal heterojunctions. Nature Mater. 4, 383–387 (2005). 15852019 10.1038/nmat1377

[b18] Kang M. S., Butsch A. & Russell P. S. J. Reconfigurable light-driven opto-acoustic isolators in photonic crystal fiber. Nature Photon. 5, 549–553 (2011).

[b19] Śmigaj W. . Magneto-optical circulator designed for operation in a uniform external magnetic field. Opt. Lett. 35, 568–570 (2010). 20160820 10.1364/OL.35.000568

[b20] Inoue M. . Magnetophotonic crystals. J. Phys. D 39, R151–R161 (2006).

[b21] Ghosh S. . Adhesively bonded Ce:YIG/SOI integrated optical circulator. Opt. Lett. 38, 965–967 (2013). 23503275 10.1364/OL.38.000965

[b22] Shoji Y. & Mizumoto T. Magneto-optical non-reciprocal devices in silicon photonics. Sci. Technol. Adv. Mater. 15, 014602 (2014). 27877640 10.1088/1468-6996/15/1/014602 PMC5090601

[b23] Wang Z. & Fan S. Optical circulators in two-dimensional magneto-optical photonic crystals. Opt. Lett. 30, 1989–1991 (2005). 16092242 10.1364/ol.30.001989

[b24] Kono N., Kakihara K., Saitoh K. & Koshiba M. Nonreciprocal microresonators for the miniaturization of optical waveguide isolators. Opt. Express 15, 7737–7751 (2007). 19547103 10.1364/oe.15.007737

[b25] Pintus P., Di Pasquale F. & Bowers J. E. Integrated TE and TM optical circulators on ultra-low-loss silicon nitride platform. Opt. Express 21, 5041–5052 (2013). 23482037 10.1364/OE.21.005041

[b26] Jalas D., Petrov A., Yu. & Eich M. Optical three-port circulators made with ring resonators. Opt. Lett. 39, 1425–1428 (2014). 24690804 10.1364/OL.39.001425

[b27] Tien M.-C., Mizumoto T., Pintus P., Kromer H. & Bowers J. E. Silicon ring isolators with bonded nonreciprocal magneto-optic garnets. Opt. Express 19, 11740–11745 (2011). 21716405 10.1364/OE.19.011740

[b28] Chang L. . Parity–time symmetry and variable optical isolation in active–passive-coupled microresonators. Nature Photon. 8, 524–529 (2014).

[b29] Peng B. . Parity-time-symmetric whispering-gallery microcavities. Nature Phys. 10, 394–398 (2014).

[b30] Armani D. K., Kippenberg T. J., Spillane S. M. & Vahala K. J. Ultra-high-Q toroid microcavity on a chip. Nature 421, 925–928 (2003). 12606995 10.1038/nature01371

[b31] Yang L., Carmon T., Min B., Spillane S. M. & Vahala K. J. Erbium-doped and Raman microlasers on a silicon chip fabricated by the sol-gel process. Appl. Phys. Lett. 86, 091114 (2005).

[b32] Fan H., Hua S., Jiang X. & Xiao M. Demonstration of an erbium-doped microsphere laser on a silicon chip. Laser Phys. Lett. 10, 105809 (2013).

[b33] Kippenberg T. J., Spillane S. M. & Vahala K. J. Modal coupling in traveling-wave resonators. Opt. Lett. 27, 1669–1671 (2002). 18033330 10.1364/ol.27.001669

[b34] Wen J. . Modeling of On-Chip Optical Nonreciprocity with an Active Microcavity. Photonics. 2, 498–508 (2015).

[b35] Lei F., Peng B., Özdemir Ş. K., Long G. L. & Yang L. Dynamic Fano-like resonances in erbium-doped whispering-gallery-mode microresonators. Appl. Phys. Lett. 105, 101112 (2014).

[b36] Shi Y., Yu Z. & Fan S. Limitations of nonlinear optical isolators due to dynamic reciprocity. Nature Photon. 9, 388–392 (2015).

[b37] Bulota F., Belanger P., Leduc M., Boudoux C. & Godbout N. Pseudo-circulator implemented as a multimode fiber coupler. Proc. SPIE 9744, 974412 (2016).

[b38] Hua S., Wen J., Jiang X., Hua Q., Jiang L. & Xiao M. Demonstration of a Chip-based Nonlinear Optical Isolator. arXiv:1606.04400 (2016). 10.1038/ncomms13657 PMC5133705 27886189

[b39] Zheng Y., Yang J., Shen Z., Cao J., Chen X., Liang X. & Wan W. Optically induced transparency in a micro-cavity. Light Sci. Appl. 5, e16072 (2016). 10.1038/lsa.2016.72 PMC6059932 30167162

