# Supplementary material for: On-Chip Optical Nonreciprocity Using an Active Microcavity
Source: Sci Rep. 2016 Dec 13;6:38972. doi: 10.1038/srep38972 (PMC5154192; doi:10.1038/srep38972)
Supplement: Supplementary Information [file srep38972-s1.pdf]

Supplementary Information

On-Chip Optical Nonreciprocity Using an Active Microcavity

Xiaoshun Jiang[1,*], *Chao Yang*[1], *Hongya Wu*[1], *Shiyue Hua*[1], *Long Chang*[1], *Yang Ding*[1], *Qian Hua*[1], *Min Xiao*[1,2]

[1]*National Laboratory of Solid State Microstructures, College of Engineering and Applied Sciences, and School of Physics, Nanjing University, Nanjing 210093, China.*

[2]*Department of Physics, University of Arkansas, Fayetteville, Arkansas 72701, USA.*

[*]*Correspondence and requests for materials should be addressed to X.J. (email: jxs@nju.edu.cn).*

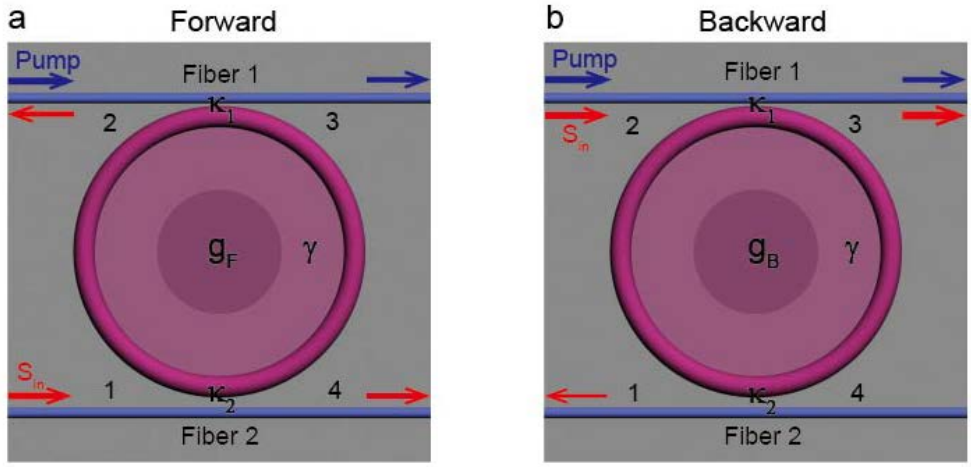

Figure S1. **On-chip optical isolation and circulation using only one active whispering-gallery-mode (WGM) microtoroid resonator.** The pump field is used to produce an effective gain (g_F in the forward propagation and g_B in the backward) for the signal wave through optically pumping doped erbium-doped ions. κ_1 and κ_2 represent the coupling strengths of toroid-fiber 1 and toroid-fiber 2, respectively. Light asymmetric transports are examined by measuring transmittance spectra in the forward (*a*) and backward (*b*) propagation configurations. s_{in} stands for the amplitude of the input signal field.

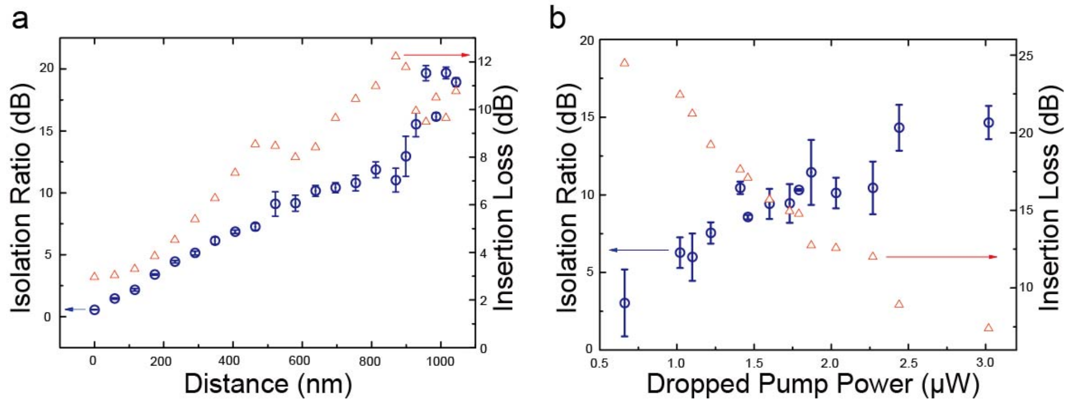

Figure S2. The measured insertion losses in correspondence to the two isolation measurements shown in Figure 3 in the main text. (*a*) The insertion loss increases along with raising the separation distance between the microcavity and fiber 2. The fixed parameters are: $\kappa_1 = 2\pi \times 0.23$ GHz and the signal power of 291 nW. **(*b*)** The insertion loss decreases when increasing the dropped pump power. The fixed parameters are: $\kappa_1 = 2\pi \times 0.24$ GHz, $\kappa_2 = 2\pi \times 1.15$ MHz, and the signal power of 291 nW.

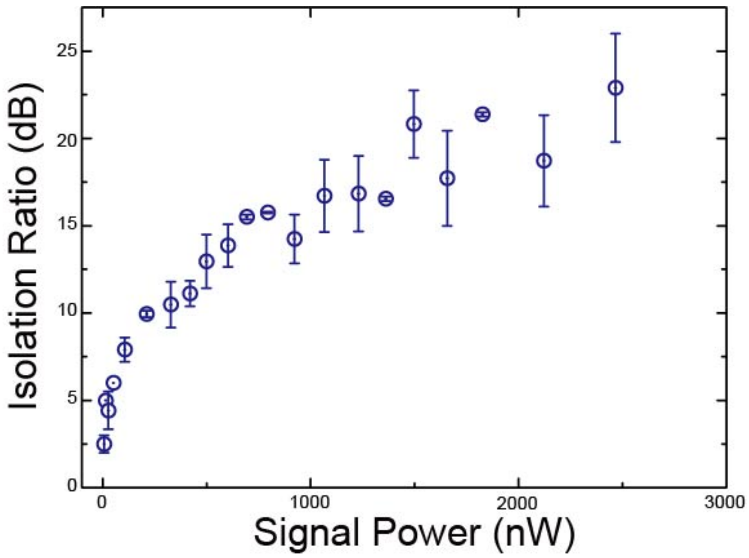

Figure S3. Optical isolation performance of the device versus the signal power. The fixed parameters are: $\kappa_i = 2\pi \times 2.5$ GHz, $\kappa_1 = 2\pi \times 107$ MHz, $\kappa_1 = 2\pi \times 4.3$ MHz and the dropped pump power of 52.1 μW.

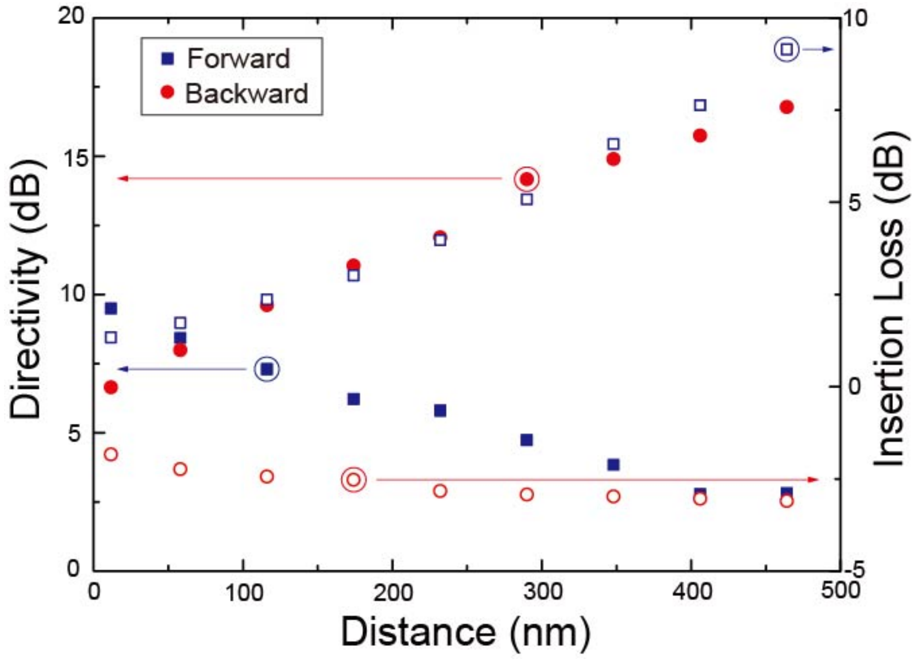

Figure S4. The trends of the forward and backward insertion losses versus the separation distance between the microresonator and the fiber taper 2. In correspondence to the measured directivities shown in Figure 4b in the main text. The fixed parameters are: $\kappa_i = 2\pi \times 0.47$ GHz, the input signal power of 312 nW and the dropped pump power of 6.60 μW.

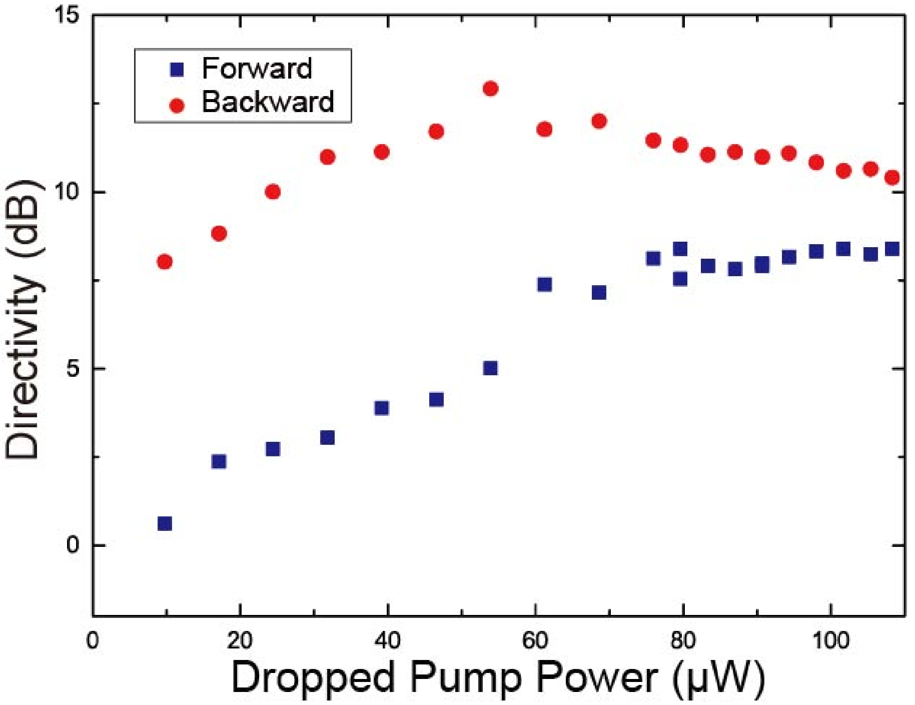

Figure S5. Optical pseudo-circulation performance of the system as a function of the dropped pump power by fixing all other parameters. The data are taken with: $\kappa_i = 2\pi \times 0.46$ GHz, $\kappa_i = 2\pi \times 9$ MHz and the signal power of 312 nW.

Supplementary Notes

Theoretical Model. A simple theoretical model based upon the coupled-mode theory[1,2] was developed to describe the signal dynamics and solved under the steady-state condition. Equation 1 given in the main text can be easily computed numerically. In the numerical calculations, even when the back-scattering effect[3] is included, good agreements are still reachable between theoretical predictions and experimentally measured results. In a simplified theoretical model (to reveal the main underlying physics), for the forward propagation configuration (Fig. S1*a*), the signal transmissions at ports 2 and 4 are described by

$$\begin{aligned}
&\left(i\Delta\omega+\frac{g_0}{2\left(1+\left|a_F/a_s\right|^2\right)}-\frac{\gamma+\kappa_1+\kappa_2}{2}\right)a_F+\sqrt{\kappa_2}S_{in}=0,\\
&s_2^F=\sqrt{\kappa_1}a_F,\\
&s_4^F=S_{in}-\sqrt{\kappa_2}a_F;
\end{aligned}\tag{S1}$$

while for the backward propagation configuration (Figure S1*b*), the signal transmissions at ports 1 and 3 are governed by

$$\begin{aligned}
&\left(i\Delta\omega+\frac{g_0}{2\left(1+\left|a_B/a_s\right|^2\right)}-\frac{\gamma+\kappa_1+\kappa_2}{2}\right)a_B+\sqrt{\kappa_1}S_{in}=0,\\
&s_1^B=\sqrt{\kappa_2}a_B,\\
&s_3^B=S_{in}-\sqrt{\kappa_1}a_B.
\end{aligned}\tag{S2}$$

Here, a_F and a_B denote the signal-field amplitudes inside the cavity for the forward and backward propagation configurations, respectively; $\Delta\omega=\omega-\omega_0$ is the cavity frequency detuning; g_0 represents the gain as $a_{F,B}=0$; and a_s stands for the

gain-saturation threshold. The forward effective gain takes the form of $g_F = \frac{1}{2}(\mathrm{g'}_F - \gamma - \kappa_1 - \kappa_2)$ and the backward effective gain is $g_B = \frac{1}{2}(\mathrm{g'}_B - \gamma - \kappa_1 - \kappa_2)$ with $g'_{F,B} = g_0 / (1 + \left|\frac{a_{F,B}}{a_s}\right|^2)$, where $g'_{F,B}$ is the real gain provided by optically pumping the doped erbium ions inside. These two sets of coupled equations (**S1** and **S2**) provide the starting point for us to examine whether the signal transport lies in the reciprocal or nonreciprocal region.

Optical Isolation. In the case of optical isolation, we are interested in the quantity of ***isolation ratio*** (IR), which is defined as

$$\text{Isolation Ratio (dB)} \equiv 10 \times \log_{10} \frac{\text{Maximum of } T_2^F}{\text{Maximum of } T_2^B}, \tag{S3}$$

with the normalized signal output transmittances at ports 2 and 1 given by

$$T_2^F = \left|\frac{s_2^F}{S_{in}}\right|^2, \text{ and } T_1^B = \left|\frac{s_1^B}{S_{in}}\right|^2. \tag{S4}$$

Isolation ratio is an important concept for quantifying and characterizing the isolation performance of a nonreciprocal device. A positive IR implies more output transmission in the forward propagation configuration than in the backward direction; while the negative IR means the opposite outcome. Despite being a simple architecture, the current scheme contains several adjustable degrees of freedom (i.e. γ, κ_1, κ_2 the input signal power, the dropped pump power, etc.) for systematic manipulations. On the one hand, these degrees of freedom offer a broad range of the parameter space to tune the achievable IRs. On the other hand, such flexibilities result

in the difficulty and complexity in the experimental implementations. To reduce the experimental complexity, in practice we can choose one degree of freedom as a variable by making all the others constant. By applying this strategy, in the current work we have carefully studied the isolation performance as a function of either κ_2 (Figure 3a), the dropped pump power (Figure 3b), or the input signal power (Figure S3). Although the one-cavity configuration makes the scheme elegant and simple, the gain-saturation nonlinearity actually brings rich physics into this system. Full theoretical evaluations with numerical simulations on the optical nonreciprocity of the device has been published elsewhere[2]. Further experimental results on optical isolation, especially on the insertion losses, will be continued below.

Optical Pseudo-Circulation (or Bidirectional Transmission). In the present work, we choose ports 1, 2 and 3 (Figure S1) to construct a three-port pseudo-circulator (or bidirectional transmission). The power flow direction is determined by coupling strengths. For simplicity, throughout the presentation (including the main text) we mainly concentrate on the following circulation scenario: if the signal enters into port 1, it drops out from port 2; but if the signal is launched from port 2, it does not come out of port 1, but instead exits from port 3. This situation can be easily fulfilled with asymmetric coupling ($\kappa_1 > \kappa_2$) in our current system. In terms of the scattering matrix, the signal outputs of this three-port pseudo-circulator is described by[2]

$$\begin{pmatrix} s_1^{out} \\ s_2^{out} \\ s_3^{out} \\ s_4^{out} \end{pmatrix} = \begin{pmatrix} 0 & \frac{\sqrt{\kappa_1\kappa_2}}{i\Delta\omega + g_B} & 0 & 1-\frac{\kappa_2}{i\Delta\omega + g_F} \\ \frac{\sqrt{\kappa_1\kappa_2}}{i\Delta\omega + g_F} & 0 & 1-\frac{\kappa_1}{i\Delta\omega + g_B} & 0 \\ 0 & 1-\frac{\kappa_1}{i\Delta\omega + g_B} & 0 & \frac{\sqrt{\kappa_1\kappa_2}}{i\Delta\omega + g_F} \\ 1-\frac{\kappa_2}{i\Delta\omega + g_F} & 0 & \frac{\sqrt{\kappa_1\kappa_2}}{i\Delta\omega + g_B} & 0 \end{pmatrix} \begin{pmatrix} s_1^{in} \\ s_2^{in} \\ s_3^{in} \\ s_4^{in} \end{pmatrix} \qquad \text{(S5 a)}$$

$$(s_i s_j)_{i \neq j} = 0, \text{ and } \sum_i s_i \neq 0 \qquad \text{(S5 b)}$$

without taking into account backscattering. Here to generalize, we mark signal launched through fiber 2 as forward propagation and signal launched through fiber 1 as backward propagation. Equation (S5b) indicates only one port works as a signal input port. Equation (S5) shows that if the signal is injected at port 3, the output will come out of port 2 instead of port 1. The lack of directional transmission $\boxed{3 \to 1}$ implies the difference of our system from a conventional circulator. As a result, this drawback sets a limitation of the device in practical applications.

In the standard procedure, the performance of a circulator is characterized by computing the isolation ratios among different port combinations. To obtain a better quantitative characterization on the light circulation ability in our scheme, in this work we introduce the concept of ***directivity*** as another figure of merit to measure the signal-power flow in the direction of its strongest emission versus the opposite direction. By doing so, it leads to the definition of the ***forward directivity*** as

$$\text{Forward Directivity (dB)} \equiv 10 \times \log_{10} \frac{\text{Maximum of } T_2^F}{\text{Maximum of } T_3^F} \qquad \text{(S6)}$$

to characterize the power flow direction as if the signal light is incident from port 1. One can immediately deduce from Eq. **S6** that in the ideal case without the existence of the backscattering, the forward directivity shall become infinity and the signal can only come out from port 2. (Recall that in this three-port pseudo-circulator, port 4 is not relevant in the analysis.) In reality, however, due to the surface roughness, the occurrence of Mie scattering will cause part of the reflected signal to be emitted from port 3[3], which diminishes the forward directivity to be finite. In a similar way, we define the ***backward directivity***

$$\text{Backward Directivity (dB)} \equiv 10 \times \log_{10} \frac{\text{Maximum of } T_3^B}{\text{Maximum of } T_1^B} \qquad \text{(S7)}$$

to characterize the power flow direction as if the signal enters from port 2. Notice that because of the gain amplification, the signal output at port 3 can actually be larger than the original input from port 2. This point has been verified in the experimental data (see the subplot $\boxed{2 \to 3}$ of Figure 4a in the main text).

Before moving to the next section, it is worth to mention that the concept of *directivity* has comprehensive applications in the fields of microwave circuits (such as distributed amplifiers)[4,5] and acoustics. We become also aware that in the characterization of the optical fiber networks, *directivity* has been commonly used to compute the ratio of the backscattered power at the other port to the input power in optical couplers[6]. It turns out that the application of the *directivity* in our work is very useful not only for evaluating quantitatively the isolation performance of the system, but also for guiding us to quickly tune the device into its optimal circulating behavior.

Further Information on Isolation Measurements

As described in the main text and the above section, high-contrast optical nonreciprocity in this system is obtained by asymmetric geometrical coupling when the system is operating in the gain-saturation regime. In light of the suggestions given in Ref. 7, all isolation measurements have been carefully implemented with the uses of optical switches (S1, S2 and S3 depicted in Figure 1d in the main text) to change the forward and backward configurations instead of manually realigning the optical paths. For more details, please refer to the schematic diagram of the experimental apparatus (Figure 1d in the main text) and the above section. In the experiment, we have found that the ultimate isolation performance of the system is mainly limited by the power detection sensitivity of the adopted photodetectors. To be more precise, as the fiber-cavity couplings become highly asymmetric, for a given input signal power its tiny backward transmission output basically falls below the commercial photodetector's noise level and is thus buried under the noise background. As a consequence, this results in a halted backward propagation and leads to near-perfect isolation performance. Such an extreme case has been experimentally approached and shown in Figure 2c in the main text. Since the optical power for the signal light is in the range of 10 nw ~10 μW and the optical quality factor of the cavity mode is around 10^6, the thermal effect can be neglected in the silica microtoroid cavity[8]. This can further justified by the theoretical calculations as shown in Figures 2-4, in which the thermal effect is not taken into account.

In this section, we would like to continue further discussions on the isolation experiments by providing more information and results. In corresponding to Figure 3a-b given in the main text, here Figure S2 a and b supply the measured companying insertion losses in those two experiments. Note that the insertion loss is defined as

$$\text{Insertion Loss (dB)} \equiv 10 \times \log_{10} \frac{1}{\text{Maximum of transmission}}. \qquad \text{(S8)}$$

Recall Figure 3a shown in the main text that by fixing κ_1, as well as the input signal and pump laser powers, the isolation ratio increases along with enlarging the separation distance between the toroid and fiber 2. In such a case, as illustrated in Figure S2*a*, the insertion loss bears a similar trend. Specifically, the isolation ratio changes from near 0.5 dB up to 19.7 dB, and the insertion loss changes accordingly from 2.9 dB to 10.7 dB. For the situation of Figure 3b where κ_1, κ_2, and the injecting signal power are set to be constants, the isolation ratio increases from 3.0 dB up to 14.7 dB as the dropped pump power is increased. Interestingly, the insertion loss drops from 24.5 dB down to 7.4 dB in such a case (see Figure S2b). The reason for this insertion-loss reduction is obvious from the definition (**S8**) plus Eq. **S1**. That is, increasing the dropped pump power is equivalent to increase the original gain (g_0), which allows the signal to receive gradually increased amplification. As a result, the insertion loss goes downward when coupling more dropped pump power into the active microcavity.

In addition to those two isolation experiments (depicted in Figure 2 a and b), we have also carried out another measurement for optical isolation as a function of the input

signal power by setting all other parameters to be constants with the use of the second sample. The experimental data are plotted in Figure S3, which illustrates that the isolation ratio increases as coupling more signal intensity into the system. This change can be easily understood from the theoretical discussions presented above. That is, the difference of the effective gain between the forward and backward directions increases with the increase of the signal power. Using the theoretical model developed above and adding certain experimental details, we can quantitatively explain all the phenomena measured in Figures S2 and S3.

Let us make a short summary on the discussed on-chip optical isolation experiments. From those implementations, it is now evident that the present microscale optical structure, compatible with the current COMS technology, is suitable for on-chip ultrasensitive, variable optical asymmetric transmission with the isolation ratio up to near infinity at the optical communication wavelength. The demonstrated optical isolation works in a broad operating scope for the input signal power ranging from ~10 nW up to ~10 μW. By manipulating the system parameters, the one-way light transport can be easily reversed for the potential application of an on-chip optical switch. More importantly, the exhibited sufficiently low insertion loss (for the isolation ratio up to 20 dB), a superior figure of merit, makes our simple scheme be a big step forward for the realization of a real, practical on-chip optical directional transmission device for integrated photonic networks. In addition, limited mainly by the power detection sensitivity of the adopted photodetectors and the insertion loss in forward transmission measurement, in the present experiment the recorded lowest

signal power of ~10 nW with an appreciable isolation ratio of about 20 dB would be very challenging without the use of a resonant structure.

Further Information on Pseudo-Circulation Measurements

In the proposed chip-based optical circulators with ring resonators and photonic crystals[9,10], both approaches are still based upon the magneto-optically induced frequency splitting between the clockwise and the counter-clockwise traveling modes. Different from those proposals, in our current work we exploit the gain-saturation nonlinearity in an active microtoroid resonator under asymmetrical coupling strengths between the microtoroid and two tapered optical fibers to achieve bidirectional transmission. Although a real optical circulator is also one type of one-way light propagation devices, it requires more constraints than a simple optical isolator. In particular, the directional circulation performance of our scheme can be further characterized via measuring the defined directivity. As a typical example, Figure 4b in the main text illustrates the trends of the forward and backward directivities as a function of the separation distance between the microcavity and fiber taper 2. It shows that as the separation distance increases, the forward directivity drops from 9.5 dB down to 2.7 dB while the backward directivity grows from 6.7 dB up to 16.8 dB. In corresponding with Figure 4b in the main text, Figure S4 further provides detailed information on the variations of the companying forward and backward insertion losses. It is apparent from Figure S4 that the insertion losses for the forward and backward transmissions abide by opposite behaviors. Specially, as the separation distance between the microcavity and fiber taper 2 increases, the forward insertion

loss grows from 1.3 dB to 9.1 dB while the backward insertion loss deceases from -1.8 dB to -3.1 dB. Such changes can be understood as follows: for the backward propagation configuration, the signal light is launched from port 2. Increasing the distance between the microcavity and fiber taper 2 effectively reduces κ_2, and thus leads to the growth of the circulating optical power inside the microcavity. This results in more transmitted optical power from port 2 to port 3 and, consequently, leads to the reduction of the insertion loss in the backward configuration. On the other hand, for the forward direction, the signal light is launched from port 1. Therefore, reducing κ_2 results in a decrease of the circulating optical power inside the microcavity, and thus leads to the reduction of the dropped signal power from port 2. As a result, the insertion loss increases in the forward configuration. The negative sign appearing in front of the backward insertion loss is a direct signature of the amplification that the signal light experiences in the path traversing from port 2 to port 3 in the backward transmission configuration.

In Figure S5, the optical bidirectional-transmission performance is also experimentally studied as a function of the dropped pump power by fixing all the other parameters. The experimental data indicate that along with increasing the dropped pump power, both forward and backward directivities get improved before merging into certain steady (saturated) value(s). The essential physics behind this measurement relies on the fact that as the pump power is gradually increased, g_0 first increases and then becomes saturated into a constant.

In the end, we want to make a few remarks on the appearance of a doublet structure shown in the subplot $\boxed{2 \rightarrow 3}$ in Figure 4a of the main text. First of all, one should be cautious that this doublet has a completely different physical origin from that due to the scattering-induced mode splitting as seen in Figure 2a-c in the main text. In fact, if one looks at these figures carefully and makes a comparison, especially on the central frequency difference between the two peaks, one would immediately realize that the mode separation shown in the subplot $\boxed{2 \rightarrow 3}$ of Figure 4a is much larger than that shown in Figure 2a-c. Moreover, we have found that the observed asymmetric doublet in the subplot $\boxed{2 \rightarrow 3}$ of Figure 4a depends on the ratio of the dropped pump and signal laser powers, and also on the scanning speed of the signal field frequency. We notice that this observation bears a similarity to the well-known effect of Fano resonance[11-13], a type of resonant scattering phenomenon where the interference between a background and a resonant scattering process generates the asymmetric line shape. Fano resonance[12] is a general wave phenomenon and examples of it can be found across various areas of physics and engineering. The essential physics in our case can be intuitively understood by the interference between the linear propagation of the signal field directly going through the fiber 1 and an instantaneous gain-amplified signal field via the active microresonator. The total suppression of the transmission spectrum at the cavity resonant frequency is a result of the balance between two traversing paths for the signal field.

Supplementary References

1. Haus, H.A. *Waves and Fields in Optoelectronics* (Prentice-Hall, New Jersey, 1984).
2. Wen J. et al. Modeling of On-Chip Optical Nonreciprocity with an Active Microcavity. *Photonics.* **2**, 498-508 (2015).
3. Kippenberg, T. J., Spillane, S. M. & Vahala, K. J. Modal coupling in traveling-wave resonators. *Opt. Lett.* **27**, 1669-1671 (2002).
4. Prasad S. N. & Li, S. M. Optimal design of low crosstalk, wideband, bidirectional distributed amplifiers. *IEEE MTT-S Int. Microw. Symp. Dig.* **1996**, 2, 847-850.
5. Collin, R. E. *Foundations for Microwave Engineering* (Wiley, New York, ed. 2, 2001).
6. Massa, N. “Fiber Optic Telecommunication” in *Fundamentals of Photonics*, C. Roychoudhuri, Ed. (SPIE, 2008).
7. Jalas, D. et al. What is - and what is not - an optical isolator. *Nature Photon*. **7**, 579-582 (2013).
8. Carmon, T., Yang, L. & Vahala, K. Dynamical thermal behavior and thermal self-stability of microcavities. *Optics Express* **12**, 4742-4750 (2004).
9. Wang, Z., Fan, S., Optical circulators in two-dimensional magneto-optical photonic crystals. *Opt. Lett.* **30**, 1989-1991 (2005).

10. Jalas, D., Petrov, A. Yu., Eich, M., Optical three-port circulators made with ring resonators. *Opt. Lett.* **39**, 1425-1428 (2014).

11. Miroshnichenko, A. E., Flach, S. & Kivshar, Y. S. Fano resonances in nanoscale structures..*Rev. Mod. Phys*. **82**, 2257-2298 (2010).

12. Fano, U. Effects of configuration interaction on intensities and phase shifts. *Phys. Rev.* **124**, 1866-1878 (1961).

13. Vittorini-Orgeas, A. A. Bianconi, From Majorana theory of atomic autoionization to Feshbach resonances in high temperature superconductors. *J. Supercond. Nov. Magn.* **22**, 215-221 (2009).
